# Supplementary material for: Tracking Animal Reservoirs of Pathogenic Leptospira: The Right Test for the Right Claim
Source: Trop Med Infect Dis. 2021 Nov 30;6(4):205. doi: 10.3390/tropicalmed6040205 (PMC8705917; doi:10.3390/tropicalmed6040205)
Supplement: Supplementary file 1 [file tropicalmed-06-00205-s001.zip › tropicalmed-1478219-supplementary.pdf]

Table S1. Details of bat samples, *Mormopterus acetabulosus* from Mauritius, used in the present study. The table includes the (a) Microscopic Agglutination Test (MAT) results and *Leptospira* molecular data obtained from the present study and the work of Dietrich et al. (2018) respectively. *RT-PCR: Real-Time Polymerase Chain Reaction, CT: Cycle Threshold, FMNH: specimen deposited in the Field Museum of Natural History.*

						**Serology results**		**Molecular results**		
**Specimen ID No.**	**ID extraction**	**Sex**	**Age**	**Date (day/month/year)**	**Site**	**MAT**	***Leptospira* serogroup (MAT titer)**	**RT - PCR**	**CT**	***Leptospira* species**
**FMNH 213456**	**152**	**F**	**A**	**14/12/10**	Palma Cave (Palma)	(-)	-	(+)		*L. borgpetersenii* related
FMNH 213457	153	F	A	14/12/10	Palma Cave (Palma)	(-)	-	(-)	-	
FMNH 213458	154	F	A	14/12/10	Palma Cave (Palma)	(-)	-	(+)		
FMNH 213459	155	M	A	14/12/10	Palma Cave (Palma)	(-)	-	(-)	-	
FMNH 213461	157	M	A	14/12/10	Palma Cave (Palma)	(-)	-	(-)	-	
FMNH 213463	159	M	A	14/12/10	Palma Cave (Palma)	(-)	-	(+)		
FMNH 213465	161	M	A	14/12/10	Palma Cave (Palma)	(-)	-	(+)		*L. borgpetersenii* related
FMNH 213466	162	M	A	14/12/10	Palma Cave (Palma)	(+)	Pyrogenes (1:200)	(+)		*L. borgpetersenii* related
FMNH 213467	163	F	A	14/12/10	Palma Cave (Palma)	(-)	-	(+)		
FMNH 213470	166	M	A	14/12/10	Palma Cave (Palma)	(-)	-	(+)		
FMNH 213471	167	M	A	14/12/10	Palma Cave (Palma)	(-)	-	(+)		
FMNH 213472	168	F	A	15/12/10	Twilight Cave (Roches Noires)	(-)	-	(-)	-	
FMNH 213475	171	F	A	15/12/10	Twilight Cave (Roches Noires)	(-)	-	(-)	-	
FMNH 213477	173	M	A	15/12/10	Twilight Cave (Roches Noires)	(-)	-	(-)	-	
FMNH 213474	170	F	A	15/12/10	Twilight Cave (Roches Noires)	(-)	-	(+)		
FMNH 213479	175	F	A	15/12/10	Twilight Cave (Roches Noires)	(-)	-	(+)		
FMNH 213481	176	F	A	15/12/10	Twilight Cave (Roches Noires)	(-)	-	(+)		
FMNH 213483	179	M	A	15/12/10	Twilight Cave (Roches Noires)	(-)	-	(-)	-	
FMNH 213485	181	F	A	15/12/10	Caverne Trois Bras (Moulin à Vent)	(+)	Panama (1:100)	(+)	42,00	
FMNH 213486	182	F	A	15/12/10	Caverne Trois Bras (Moulin à Vent)	(-)	-	(-)	-	
FMNH 213487	183	F	A	15/12/10	Caverne Trois Bras (Moulin à Vent)	(-)	-	(+)	34,00	*L. borgpetersenii* related
FMNH 213489	185	F	A	15/12/10	Caverne Trois Bras (Moulin à Vent)	(-)	-	(+)	37,00	
FMNH 213490	186	F	A	15/12/10	Caverne Trois Bras (Moulin à Vent)	(-)	-	(+)	35,00	
FMNH 213491	187	F	A	15/12/10	Caverne Trois Bras (Moulin à Vent)	(-)	-	(+)	38,00	
FMNH 213493	189	F	A	15/12/10	Caverne Trois Bras (Moulin à Vent)	(+)	Panama (1:200)	(+)	34,00	

FMNH 213494	190	F	A	15/12/10	Caverne Trois Bras(Moulin à Vent)	(+)	Panama (1:400)	(+)	35,00
FMNH 213495	191	F	A	15/12/10	Caverne Trois Bras (Moulin à Vent)	(-)	-	(+)	
FMNH 213503	199	F	A	16/12/10	Camp Thorel Cave (Camp Thorel)	(+)	Panama (1:400)	(+)	40,00
FMNH 213504	200	M	A	16/12/10	Camp Thorel Cave (Camp Thorel)	(-)	-	(+)	34,00
FMNH 213508	204	M	A	16/12/10	Camp Thorel Cave (Camp Thorel)	(+)	Panama (1:400)	(+)	37,00
